# Glutamatergic Synapse Dysfunction in *Drosophila* Neuromuscular Junctions Can Be Rescued by Proteostasis Modulation

**DOI:** 10.3389/fnmol.2022.842772

**Published:** 2022-07-15

**Authors:** Anushka Chakravorty, Ankit Sharma, Vasu Sheeba, Ravi Manjithaya

**Affiliations:** ^1^Autophagy Laboratory, Molecular Biology and Genetics Unit, Jawaharlal Nehru Centre for Advanced Scientific Research, Bangalore, India; ^2^Chronobiology and Behavioural Neurogenetics Laboratory, Neuroscience Unit, Jawaharlal Nehru Centre for Advanced Scientific Research, Bangalore, India; ^3^Neuroscience Unit, Jawaharlal Nehru Centre for Advanced Scientific Research, Bangalore, India

**Keywords:** Spinocerebellar Ataxia Type 3, *Drosophila* neuromuscular junctions, glutamatergic synapse, synapse dysfunction, synaptopathy, autophagy

## Abstract

Glutamate is the major excitatory neurotransmitter in the nervous system, and the *Drosophila* glutamatergic neuromuscular junctions (NMJs) offer a tractable platform to understand excitatory synapse biology both in health and disease. Synaptopathies are neurodegenerative diseases that are associated with synaptic dysfunction and often display compromised proteostasis. One such rare, progressive neurodegenerative condition, Spinocerebellar Ataxia Type 3 (SCA3) or Machado-Joseph Disease (MJD), is characterized by cerebellar ataxia, Parkinsonism, and degeneration of motor neuron synapses. While the polyQ repeat mutant protein ataxin-3 is implicated in MJD, it is unclear how it leads to impaired synaptic function. In this study, we indicated that a *Drosophila* model of MJD recapitulates characteristics of neurodegenerative disorders marked by motor neuron dysfunction. Expression of 78 polyQ repeats of mutant ataxin-3 protein in *Drosophila* motor neurons resulted in behavioral defects, such as impaired locomotion in both larval and adult stages. Furthermore, defects in eclosion and lifespan were observed in adult flies. Detailed characterization of larval glutamatergic neuromuscular junctions (NMJs) revealed defects in morphological features along with compromised NMJ functioning. Autophagy, one of the key proteostasis pathways, is known to be impaired in the case of several synaptopathies. Our study reveals that overexpression of the autophagy-related protein Atg8a rescued behavioral defects. Thus, we present a model for glutamatergic synapse dysfunction that recapitulates synaptic and behavioral deficits and show that it is an amenable system for carrying out genetic and chemical biology screens to identify potential therapeutic targets for synaptopathies.

## Introduction

Synapses, the terminal ends of neurons, are highly complex structures. Properly functioning synapses are critical to the integrity of neuronal networks in the brain, and any dysfunction of synapses may lead to the manifestation of neurodegenerative disorders (Lepeta et al., 2016; Bae and Kim, 2017). The term “synaptopathy” was introduced to include brain disorders arising as a result of synaptic dysfunction (Li et al., 2003). Synaptopathy of one of the major excitatory synapses, i.e., the glutamatergic synapse, has been reported in several neurodegenerative and neurodevelopmental disorders, including autism spectrum disorders (ASD), Down syndrome (DS), and intellectual disabilities (ID) (Südhof, 2008; Hussain et al., 2014; Tang et al., 2014; Volk et al., 2015). Although postsynaptic dysfunction, due to defects in α-amino-3-hydroxy-5-methyl-4-isoxazole propionic acid receptors (AMPARs), N-methyl-d-aspartate receptors (NMDARs), and metabotropic glutamate receptors (mGluRs), is well studied, the contribution of impaired presynapses in the manifestation of synaptopathies has not been well-characterized.

Spinocerebellar Ataxia Type 3 (SCA3; also known as Machado-Joseph disease, MJD) is a disease belonging to a group of progressive neurodegenerative disorders that are characterized by gait ataxia, ophthalmoplegia, and amyotrophy (Maciel et al., 1995; Paulson, 2012; McLoughlin et al., 2020). Although muscle weakness and loss of muscles are common signs of this late-onset neurodegenerative disease, the involvement of the peripheral nervous system in the progression of the disease is less understood. The pathogenesis of the disease is attributed to the expanded CAG (coding for Glutamine – Q) repeats in the coding region of the *ATXN3* gene (formerly known as the *MJD1* gene) that encodes a 42 kDa protein, ataxin-3 (or ATXN3). ATXN3 is a deubiquitinating enzyme that preferentially binds to and cleaves long polyubiquitin chains. It harbors multiple ubiquitin-interacting motifs (UIMs) and a catalytic Josephin domain (Nicastro et al., 2010). Non-pathogenic ATXN3 contains polyQs that may range from ~12 to 43 repeats in length, which could increase beyond 60, in the case of the pathogenic form. PolyQ-expanded ATXN3 has been reported to aggregate in the nuclei of cultured cells and in neurons of various model organisms (Chai et al., 1999; Colomer Gould, 2005; Bichelmeier et al., 2007; Koeppen, 2018). These reports are consistent with patient sample data, which show the formation of inclusions in the brains of patients with MJD (Schmidt et al., 1998; Goti et al., 2004). Although the protein is ubiquitously expressed in several cell types, the mutant form of ATXN3 specifically affects some regions of the brain (Toonen et al., 2018). Extensive research has been done to understand the pathophysiology of MJD in the central nervous system (Alves et al., 2008; Nguyen et al., 2013; Nóbrega et al., 2013; Konno et al., 2014). However, the involvement of polyQ-expanded mutant ATXN3 in the etiology of peripheral nervous system dysfunction is less explored. We investigated the contribution of the polyQ-expanded mutant ATXN3 in the pathogenesis of peripheral nervous system disorders and glutamatergic synapse dysfunction.

We took advantage of the genetic amenability of the fly *Drosophila melanogaster* to generate a model of synaptopathy by targeting the pathogenic form of ATXN3 protein, containing 78 polyQ repeats to the neuromuscular junction. We observed morphological and functional defects in the synapses of motor neurons, which correlate with behavioral deficits observed both in the larval as well as adult stages of flies. As with many other polyQ disorders, we also observed proteostasis impairment in the motor neurons of flies. PolyQ aggregates have been shown to contribute to the pathology of several neurodegenerative disorders by impairment of the autophagy pathway and genetic or pharmacological induction of the pathway might help restore proteostatic imbalance. Here, we show that, with the aid of tissue-specific motor neuron overexpression of one of the core autophagy proteins, Atg8a, there is significant improvement in the behavioral and functional defects of glutamatergic synapses *in vivo*.

## Materials and Methods

### Fly Husbandry

*Drosophila melanogaster* was reared on standard cornmeal agar supplemented with yeast, at 25°C with 12:12 h light-dark cycle. All crosses were set up at 25°C. The following stocks were obtained from Bloomington *Drosophila* Stock Center (BDSC): *UAS-MJDtrQ27* (8149), *UAS-MJDtrQ78* (8150), *UAS-mCherry-Atg8a* (37750), and *D42-Gal4* (8816).

### Larval NMJ Fillet Preparation and Immunohistochemistry

Third instar larvae were dissected in HL3 buffer (70 mM NaCl, 5 mM KCl, 20 mM MgCl_2_, 5 mM trehalose, 115 mM sucrose, 5 mM HEPES, 10 mM NaHCO_3_, pH 7.2), internal organs were removed, and muscle fillet was prepared. The dissected fillets were fixed in 4% paraformaldehyde for 20 min and incubated in 0.1% PBT (0.1% Triton X-100 in PBS) for 30 min. Blocking was done in 0.2% PBTB (0.2% BSA in PBT) for 1 h, followed by incubation in 2% PBTN (2% normal goat serum in PBTB) for 30 min. After blocking, samples were kept for overnight primary antibody incubation at 4°C. After several washes and blocking in 2% PBTN, samples were incubated in secondary antibody at room temperature for 2 h. Following washes, the samples were mounted in Vectashield (Vector Laboratories). The following antibodies and their dilutions were used: mouse anti-Brp at 1:200 (Developmental Studies Hybridoma Bank), mouse anti-synaptotagmin at 1:50 (Developmental Studies Hybridoma Bank), mouse anti-HA at 1:200 (Invitrogen), and rabbit anti-GABARAP at 1:200 (Abcam). Secondary antibodies used were goat anti-mouse Atto 633 at 1:1,000 (Sigma-Aldrich), goat anti-mouse Atto 550 at 1:1,000 (Sigma-Aldrich), goat anti-rabbit Atto 633 at 1:1,000 (Sigma-Aldrich), goat anti-rabbit Atto 550 at 1:1,000 (Sigma-Aldrich), FITC-HRP, and Dylight-HRP at 1:200 (Jackson ImmunoResearch Laboratories, Inc.). Images for morphometric analysis were acquired on an LSM 880 confocal microscope using Zen software (Zeiss). Images for synaptotagmin intensity quantification were acquired on a DeltaVision microscope using SoftWorx software (GE Healthcare). For comparison of protein levels between different genotypes, all the samples were processed on the same day under identical conditions. Z-stacks were acquired with 0.25 μm spacing.

### Larval Ventral Nerve Cord Preparation and Immunohistochemistry

Brains from third instar larvae were dissected in ice-cold PBS and fixed in 4% paraformaldehyde at room temperature for half an hour. Following fixation, the samples were washed three times for 10 min with 0.5% PBT (0.5% Triton X-100 in PBS). After washes, the larval brains were blocked using 10% horse serum (prepared in 0.5% PBT). The brains were incubated overnight in primary antibody at 4°C. Following washes (four times, 10 min each using 0.5% PBT), the brains were incubated in secondary antibody at room temperature. Finally, the samples were washed, mounted in a glycerol-containing medium with DAPI, and imaged using a Zeiss LSM 880 confocal microscope and a 63X/1.4 DIC oil immersion objective using Zen software (Zeiss). The following antibodies were used: mouse anti-HA at 1:200 (Invitrogen) and FITC-HRP at 1:200 (Jackson ImmunoResearch Laboratories, Inc.). The secondary antibody used was goat anti-mouse 546 (1:3,000, Invitrogen).

### Analysis of Larval NMJ

For quantification of bouton numbers, NMJs of muscle 6/7 of hemisegment A2 were acquired with a 40X objective using a laser-scanning confocal microscope (Zeiss LSM 880). The respective muscle area was acquired with a 20X objective. The number of boutons was normalized to the muscle area. For quantification of the bouton area and branch length, NMJs of muscle 6/7 of hemisegment A4-A6 were used. Quantification of synaptotagmin intensity was done on NMJs of muscle 4 of hemisegment A2. Manual quantification of the number of boutons, bouton area, NMJ branch length, and satellite boutons was done on maximum intensity projection (MIP) of images using ImageJ software (National Institutes of Health, NIH). Quantification of Brp puncta was done using Particle Analysis tool in ImageJ on thresholded images in NMJs of muscle 6/7 of hemisegment A2-A4. Genotype-blind quantification was carried out for all the acquired images.

### Behavioral Assays

#### Larval Locomotion Assay

Third instar larvae of desired genotypes were collected, washed in distilled water, and subjected to a locomotion assay on 1% charcoal agar plates. Four larvae were placed at the center of the plate, and recordings were carried out for 2 min per video at ambient room temperature (25°C). Eight such videos (thus, 32 larvae per genotype) were captured at a frame rate of 30 fps and were uncompressed and processed for further analysis using VirtualDub 1.10.4. Analysis was done using WrmTrck plugin of ImageJ with the following parameters: rolling ball radius – 0.7, minimum object area – 10, maximum object area – 400, maximum velocity – 10, maximum area change – 200, minimum track length – 500, the threshold for turn – 2, size of a bin for speed histogram – 0, frames per second – 30, thresholding method – Otsu. Analysis for coiling behavior, bends and turns, and orientation counts were done using FIMTrack version 2 (Risse et al., 2017). Representative figures were made using iPython (Jupyter Notebook).

#### Eclosion Assay and Wing Defect Phenotype Assay

Crosses were set up for indicated genotypes and maintained at a 12:12 h light-dark cycle. Eclosion patterns were studied from four replicate vials, containing ~5 ml of cornmeal agar and housing > 60 pupae per vial. All the vials were monitored till eclosion, and the number of flies eclosing was counted every 6 h for 4 consecutive days. Following eclosion, the number of flies with different wing phenotypes (fully expanded, or degenerated, or half expanded) was counted. Images for wing phenotypes were captured using an SZX16 Olympus stereomicroscope.

#### Activity Counts

Male flies were collected post-eclosion. Then, two three-day-old flies were loaded onto activity tubes with cornmeal food and monitored using *Drosophila* Activity Monitors (DAM, TriKinetics). Recordings were done under 12:12 h light-dark cycles (light intensity, ~250lx) at a constant temperature of 25°C. Collected data were binned (20-min bin length) using DAM File Scanner and the activity counts were analyzed using Microsoft Excel.

#### Adult Lifespan Assay

To perform the lifespan assay, freshly eclosed males and females were collected and aged for 3 days, separated according to sex, and transferred into 4 replicate vials containing cornmeal agar food in groups of 15 flies/vial. Thus, 60 male and female flies of each genotype were assayed. The flies were transferred to fresh media every alternate day, and longevity was estimated by counting the number of flies alive in each vial. The assay was performed for 28 days.

### Statistical Analysis

Statistical analysis was done using GraphPad Prism (version 8.4.2) and Statistica 7. Statistics employed for quantification are described in legends of respective figures and in the results section.

## Results

### Expression of Mutant Q78 in Motor Neurons Causes Locomotory Deficits in Larvae

**Figure 1:**
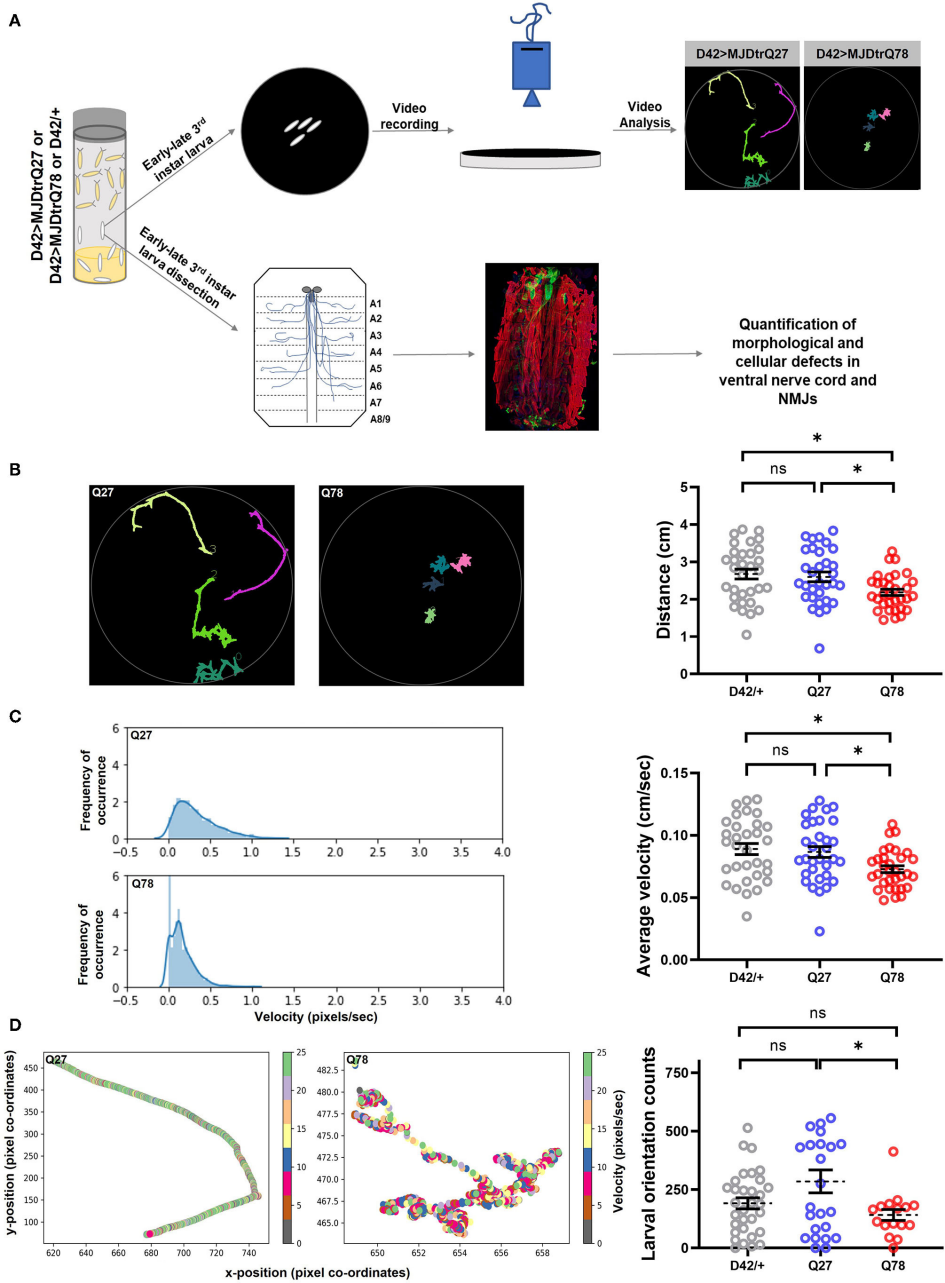
Q78 expression in motor neurons leads to larval locomotion defect. **(A)** A flow chart of approaches to characterize behavioral and cellular defects upon expression of mutant MJDtrQ78. Third instar larvae expressing non-pathogenic Q27 or pathogenic Q78 along with driver control D42/+ were subjected to a larval locomotion assay and simultaneously assessed for phenotypic and functional defects in NMJs. **(B)** Path diagrams for Q27 and Q78 larvae monitored in 1% charcoal agar plates and quantification of the total distance traversed by the driver-only control larvae (D42/+) vs. non-pathogenic Q27 and pathogenic Q78 larvae. *n* = 32 larvae; one-way ANOVA; *post-hoc* Tukey's multiple comparison test; ns, non-significant; *p < 0.05; error bars represent mean ± SEM. **(C)** Representative images of the velocity distribution of non-pathogenic Q27 and pathogenic Q78 larvae and quantification of the average velocity of larvae. *n* = 32 larvae; one-way ANOVA; *post-hoc* Tukey's multiple comparison test; ns, non-significant; *p < 0.05; error bar represents ± SEM. **(D)** A representative scatter plot showing instantaneous velocity per coordinate for non-pathogenic Q27 and pathogenic Q78 larvae. The number of times the larvae traversed linearly in the arena without reorientation/coiling was plotted as orientation counts. *n* = 32 larvae; one-way ANOVA; *post-hoc* Tukey's multiple comparison test; ns, non-significant; *p < 0.05; error bar represents mean ± SEM.

Previous studies have revealed an intricate association of locomotory deficits with synaptic dysfunction, such as decrease in synaptic connections, changes in the presynaptic and postsynaptic proteomes, and reduced neurotransmitter release (Mhatre et al., 2014; Kashima et al., 2017). To examine the effect of polyglutamine repeats of ATXN3 protein on the peripheral nervous system and glutamatergic synapses at the gross behavioral level, we made use of the GAL4-UAS system (Brand and Perrimon, 1993). Fly motor neurons were targeted using the *D42-Gal4* driver, the expression of which is restricted to motor neurons and interneurons within the larval nervous system and to motor neurons in the adult nervous system (Yeh et al., 1995; Gustafson and Boulianne, 1996). We expressed 78 polyQ repeats flanked upstream by 12 amino acids and downstream by 43 amino acids of ATXN3 protein containing an N-terminal HA tag (MJDtrQ78, henceforth called Q78). Non-pathogenic ATXN3 containing 27 polyQ repeats (MJDtrQ27, henceforth called Q27) served as the control. The Gal4 driver served as the parental driver control. Third instar larvae were subjected to a larval locomotion assay with various parameters in 1% charcoal agar plates, including distance traveled in the arena and average velocity, and were quantified as a surrogate readout for proper functioning of motor neurons (Figure 1A). We observed defects in larval locomotion on the expression of Q78 in motor neurons.

We first quantified the total path traversed by the larvae and observed a significant reduction in path length traveled by Q78 expressing larvae as compared to both Q27 and D42/+ control larvae [genotype, *F*_(2, 93)_ = 5.005, *p* = 0.0086 ANOVA; D42/+ vs. Q27 (*p* = 0.8902), D42/+ vs. Q78 (*p* = 0.0114), Q27 vs. Q78 (*p* = 0.0392) Tukey's multiple comparison] (Figure 1B). Furthermore, we assessed the average velocity of locomotion and observed a similar significant reduction in velocity of Q78 expressing larvae as compared to both the controls [genotype, *F*_(2, 93)_ = 5.004, *p* = 0.0086, ANOVA; D42/+ vs. Q27 (*p* = 0.9037), D42/+ vs. Q78 (*p* = 0.0118), Q27 vs. Q78 (*p* = 0.0374), Tukey's multiple comparison] (Figure 1C). The larval orientation counts were also reduced upon the expression of mutant Q78; however, this parameter was not significantly different from the D42/+ control [genotype, *F*_(2, 69)_ = 3.773, *p* = 0.0279, ANOVA; D42/+ vs. Q27 (*p* = 0.1137), D42/+ vs. Q78 (*p* = 0.6095), Q27 vs. Q78 (*p* = 0.0304), Tukey's multiple comparison] (Figure 1D). Other features of locomotion, such as the number of linear crawling events (go-phase counts), number of coiling events, and number of reorientation counts (left and right bends), were, however, unchanged as compared to controls (Supplementary Figures 1A–C). These results show that the expression of expanded polyQ repeats of ATXN3 in motor-neurons leads to locomotory deficits at the larval stage.

### Expression of Mutant Q78 in Motor Neurons Leads to Various Behavioral Defects in Adult Flies

**Figure 2:**
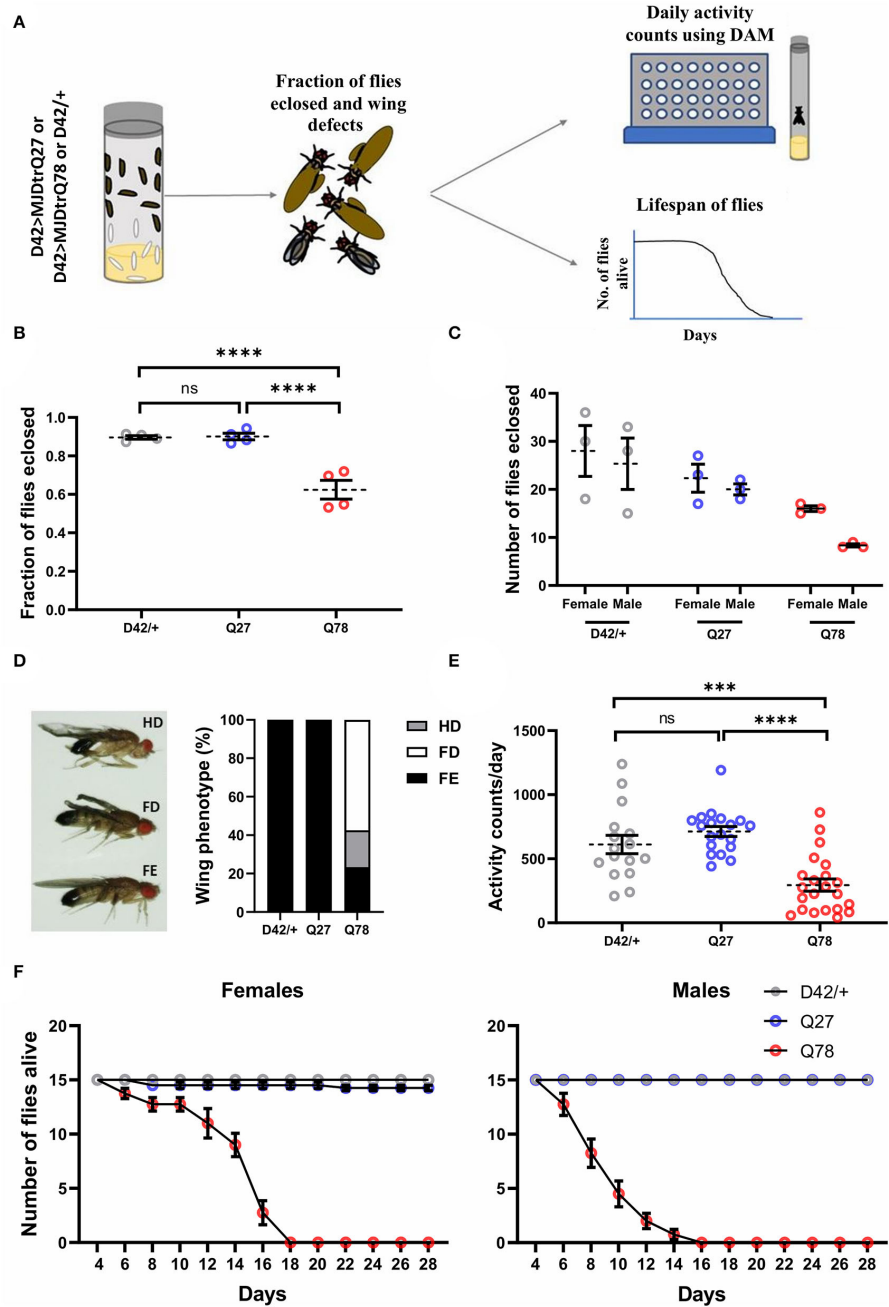
Eclosion, locomotion, and lifespan are affected in adult flies on the expression of Q78 in motor neurons. **(A)** A flow chart depicting phenotypes characterized in adult flies expressing non-pathogenic Q27 or pathogenic Q78 in motor neurons using the *D42-Gal4* driver. A fraction of adult flies that successfully emerged from pupae, wing phenotypes, locomotion, and adult lifespan was assayed. **(B)** Quantification of fraction of flies eclosed. n > 280 pupae from 4 vials; 3x2 contingency test followed by Fisher's Exact test; ****p < 0.0001; ns, non-significant; error bars represent mean ± SEM. **(C)** Quantification of the number of male and female flies eclosed. *n* ≥ 70 flies. **(D)** Images depicting wing phenotypes of adult flies expressing pathogenic Q78 under *D42-Gal4* and quantification of the percentage of wing phenotypes observed. *n* ≥ 47 flies; FD, full degeneration; HD, half degeneration; FE, fully expanded. **(E)** Quantification of activity counts per day. *n* ≥ 16 flies per genotype; one-way ANOVA; *post-hoc* Tukey's HSD; ****p < 0.0001; ***p < 0.001; ns, non-significant; error bars represent mean ± SEM. **(F)** Survivorship plots showing the number of flies alive across 28 days. *n* = 60 flies for both males and females; error bars depict mean ± SEM.

Following defects observed at the larval stages, we explored how the expression of mutant Q78 protein affects adult flies. Previous studies have shown that the expression of Q78 protein in *Drosophila* brain using the pan-neuronal driver *Elav-Gal4* leads to eclosion, wing, locomotor, and lifespan defects (Singh et al., 2014). Since this driver has a very broad expression, it is not possible to pinpoint the spatial and temporal patterns of the defects obtained. We maintained a restricted tissue target to examine the impact of Q78 mutant protein in motor neurons on adult fly behaviors. We studied various behavioral parameters, such as eclosion, wing, locomotion, and lifespan, in these flies (Figure 2A). For these experiments, the D42/+ served as the parental control. Upon quantification, we observed that motor-neuron-driven Q78 expression in adult flies led to defects or failure of eclosion, while both the control flies exhibited eclosion of > 90% [χ_(df = 2)_ = 96.51, *p* = 0.000, D42 vs. Q78 (*p* < 0.00001), D42 vs. Q27 (*p* = 0.7504), Q27 vs. Q78 (*p* < 0.00001)] (Figure 2B). Furthermore, out of all the eclosed flies, the proportion of female flies was higher in comparison to male flies (Figure 2C), whereas controls showed the expected 1:1 sex ratio. These results suggest higher male susceptibility toward Q78 pathology. This result is in line with available literature in humans, which report gender bias in the progression of many neurodegenerative conditions (Hanamsagar and Bilbo, 2016; Piscopo et al., 2021). We also observed wing defects in eclosed flies on the expression of Q78 protein. The majority of the flies had completely degenerated wings followed by a small fraction of flies, showing half degenerated wings and very few with fully expanded wings. Such wing defects were absent in control flies, wherein all the wings were fully expanded (Figure 2D). Locomotion in adult Q78 flies was also assessed using *Drosophila* Activity Monitors (DAM). Q78-expressing flies had significantly lower activity counts per day as compared to both control flies [genotype, *F*_(2, 54)_ = 18.76, *p* < 0.0001, ANOVA; D42/+ vs. Q27 (*p* = 0.3945), D42/+ vs. Q78 (*p* = 0.0003), Q27 vs. Q78 (*p* ≤ 0.0001), Tukey's multiple comparison] (Figure 2E). Finally, we assessed the survival of male and female Q78-expressing flies. We observed that both male and female flies had a comparatively shorter lifespan as compared to control flies. Moreover, we observed similar sex-based differences, wherein female flies showed a slightly longer lifespan in comparison to male flies (Figure 2F). Overall, these results showed that, upon expression of mutant Q78 protein in motor neurons, there are behavioral defects at both larval as well as adult stages of flies.

### Expression of Q78 in Motor Neurons Leads to Morphological Changes in *Drosophila* NMJs

**Figure 3:**
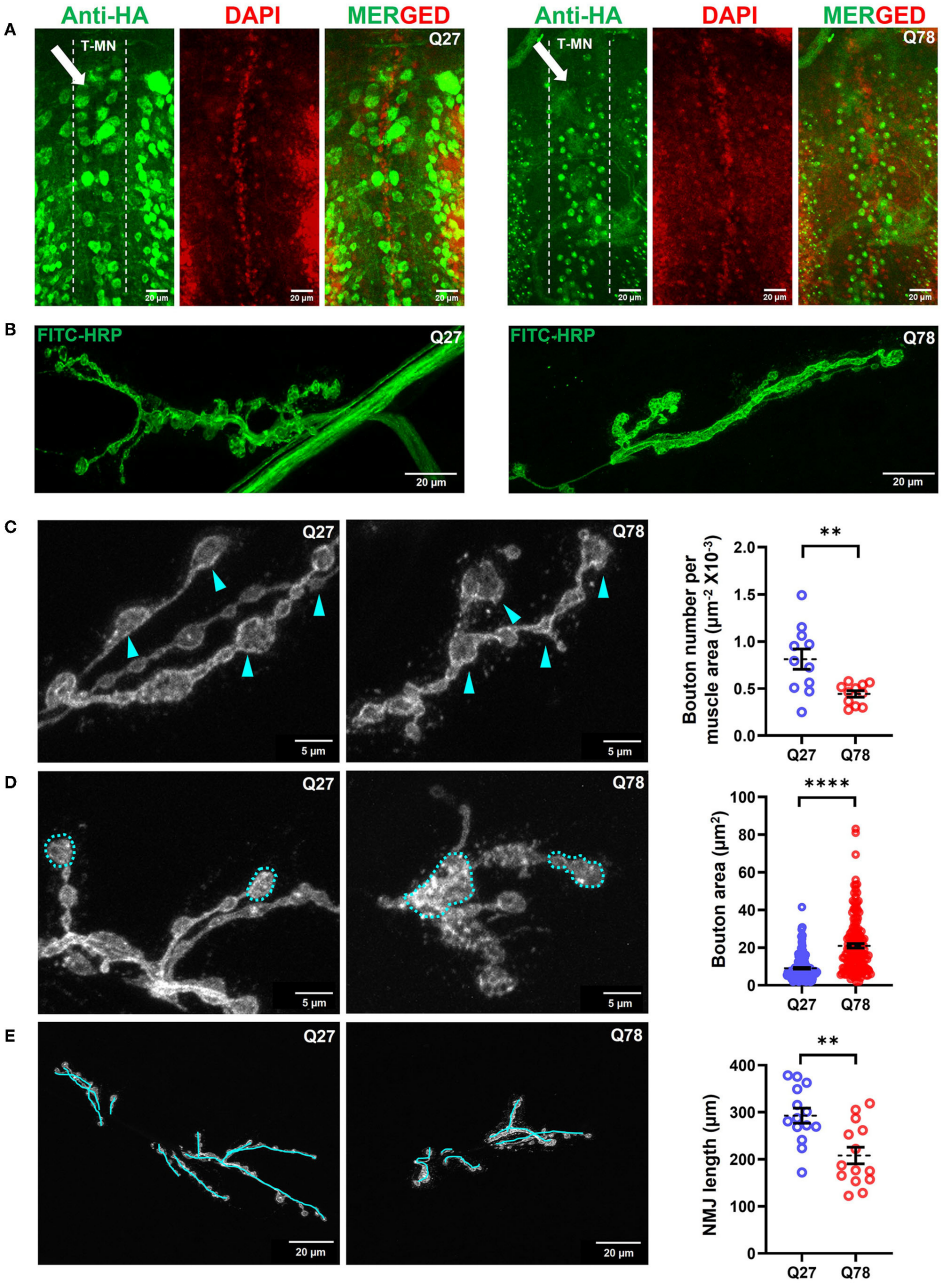
Q78 expression in motor neurons leads to multiparametric changes in the morphology of NMJs. **(A)** An image depicting the cell bodies of motor neurons in the ventral nerve cord (VNCs) of *Drosophila* larvae. An arrow depicts the alignment of the thoracic motor neuron (T-MN) cell bodies. FITC-HRP marks the neuronal membrane. HA antibody was used for staining the pathogenic or non-pathogenic ATXN3 polyQ protein. **(B)** Image depicting the NMJs of the third instar larva marked by FITC-HRP in Q27 and Q78-expressing larvae. The morphometric changes observed are further quantified. **(C)** The number of boutons per muscle area in Q27 and Q78-expressing larvae and quantification of the same. Arrowheads depict the varicosities or boutons. All quantifications were done on NMJs of muscle 6/7 of abdominal hemisegment A2. *n* > 11; Student's *t*-test; **p < 0.01. An error bar represents mean ± SEM. **(D)** The area of boutons in Q27 and Q78-expressing larvae and quantification of the same. Markings indicate the areas considered for quantification. All quantifications were done on NMJs of muscle 6/7 of abdominal segments A4-A6. *n* > 160; Student's *t*-test; ****p < 0.0001. An error bar represents mean ± SEM. **(E)** Length of NMJ branches and the main arbor in Q27 and Q78-expressing larvae and quantification of the same. Markings represent the lengths considered for quantification. All quantifications were done on NMJs of muscle 6/7 of abdominal segments A4-A6. *n* > 12; Student's *t*-test; **p < 0.01. An error bar represents mean ± SEM.

Mutant Q78 may aggregate as neuronal nuclear inclusions (NNIs), which contain ubiquitin, heat shock proteins, transcription factors, and polyQ proteins (Fujigasaki et al., 2000; Breuer et al., 2010). It may also aggregate as neuronal cytoplasmic inclusions (NCIs), which are majorly ubiquitin negative (Hayashi et al., 2003; Yamada et al., 2004). The role of either of these inclusions in the progression of MJD pathology in the peripheral nervous system is hitherto unknown. To investigate the distribution of the mutant protein expressed under *D42-Gal4* motor neuron driver *in vivo*, we dissected third instar larvae and immunostained for the truncated form of ATXN3 tagged with HA. In control Q27-expressing larvae, the non-pathogenic ATXN3 was distributed throughout the ventral nerve cord (VNC), axons, and neuromuscular junctions (NMJs, the varicosities or the presynaptic terminals of motor neurons). However, we found that Q78 expression was restricted to the cell bodies in VNC, where it accumulated as aggregates but was rarely present in the NMJs (Figure 3A, Supplementary Figures 2A,B). This result was in line with the previously observed distribution pattern of Q78 in flies using the *Elav-Gal4* driver (Lee et al., 2004).

We next assessed whether the accumulation of Q78 aggregates in the cell bodies of motor neurons led to changes in the overall morphology of glutamatergic synapses. The motor neurons MN 6/7-Ib and MNSN b/d-Is emanating from the VNC, innervate the musculature present in abdominal hemisegments 6/7 (Budnik, 1996; Menon et al., 2013). A bouton was defined as a swollen presynaptic terminal emanating from the main arbor of NMJ immunostained by FITC-HRP. The accumulation of mutant Q78 in the VNC led to characteristic morphological changes in the NMJs (Figure 3B). Structural analysis of the NMJs 6/7 showed that the number of boutons per NMJ was reduced by ~50% upon expression of mutant Q78 [t_(20)_ = 3.271, *p* = 0.0038, *t*-test] (Figure 3C). Upon expression of mutant Q78, however, we observed an ~2-fold increase in the individual bouton area, as compared to Q27 controls [t_(366)_ = 10.32, *p* < 0.0001, *t*-test] (Figure 3D). The number of branching arbors of NMJs was also decreased in the Q78 expressing motor neurons [t_(26)_ = 3.552, *p* = 0.0015, *t*-test] (Figure 3E). Moreover, we observed heterogeneity among the affected NMJs, with some exhibiting more severe defects compared to others (Supplementary Figure 2C). Altogether, these observations suggest that the expression of mutant Q78 in motor neurons led to structural changes in the glutamatergic NMJs of *Drosophila*.

### Motor Neuronal Expression of Q78 Leads to Functional Defects in Glutamatergic Synapses

Since our initial locomotion assays (Figure 1A) showed deficits in the larval locomotory behavior, we examined if the presynapses of motor neurons were affected in terms of neurotransmitter release. For this, we quantified two aspects of neurotransmitter release; the clustering of release-ready pools of synaptic vesicles (SVs) at the active zones and for the fusion of SVs.

**Figure 4:**
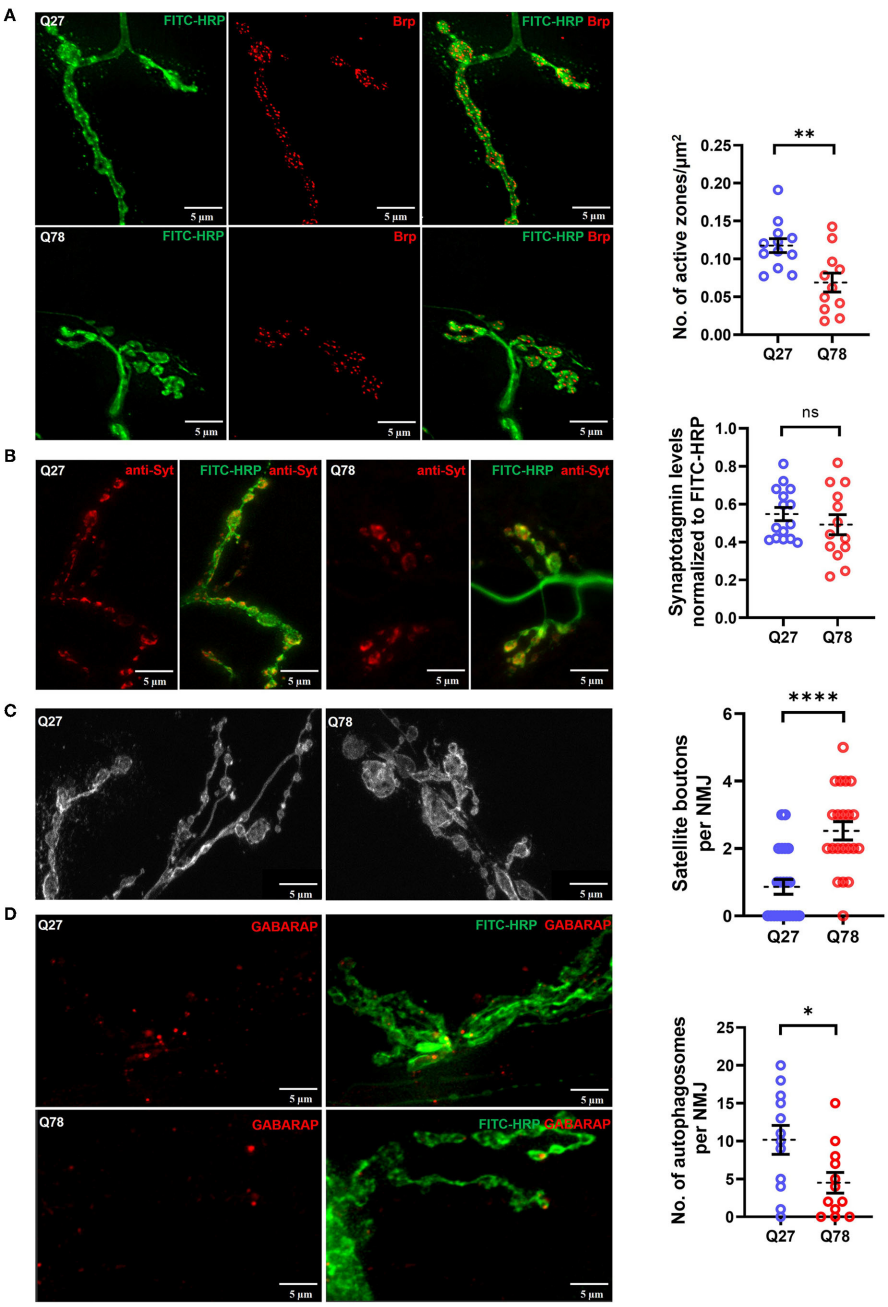
Q78 expression in larval NMJs leads to functional changes in glutamatergic synapses. **(A)** A representative image of active zones marked by anti-Brp in the NMJs of the third-instar larva marked by FITC-HRP in Q27 and Q78-expressing larvae. Quantification of the number of active zones normalized to the area of NMJ. All quantifications were done on NMJs of muscle 6/7 of abdominal segments A4-A6. *n* ≥ 12; Student's *t*-test; **p < 0.01. An error bar represents mean ± SEM. **(B)** Representative images of synaptotagmin (Syt) intensity in the third instar larval NMJ marked by FITC-HRP in Q27 and Q78-expressing larvae and quantification of Syt signal intensity in the boutons. All quantifications were done on NMJs of muscle 6/7 of abdominal segments A4-A6. *n* ≥ 15; Student's *t*-test; ns, non-significant. An error bar represents mean ± SEM. **(C)** Representative images depicting satellite boutons budding from the main boutons in NMJs with Q78 expression along with quantification of the number of satellite boutons per NMJ. All quantifications were done on NMJs of muscle 6/7 of abdominal segments A4-A6. *n* > 25; Student's *t*-test; ****p < 0.0001. An error bar represents mean ± SEM. **(D)** The figure depicts the number of autophagosomes marked by anti-GABARAP in NMJs stained by FITC-HRP in Q27- and Q78-expressing larvae. All quantifications were done on NMJs of muscle 6/7 of abdominal segments A4-A6. *n* > 12; Student's *t*-test; *p < 0.05. An error bar represents mean ± SEM.

In addition, the aberrant bouton morphology observed upon expression of Q78 led us to ask whether there were functional defects in the glutamatergic synapses as well, for which we assessed the levels of the *Drosophila* ortholog of ELKS/CAST/ERC protein called Bruchpilot (Brp) (Chou et al., 2020). Active zone sites in presynapses of *Drosophila* NMJs are marked by an increased accumulation of Brp (Van Vactor and Sigrist, 2017). Another SNARE protein involved in the fusion of the glutamate-rich vesicles is Synaptotagmin (Syt) (Paul et al., 2015; Kittel and Heckmann, 2016). We compared the levels of Brp and Syt in *Drosophila* NMJs and visualized Brp positive puncta and Syt intensity using confocal microscopy. When compared to control Q27 larvae, Q78 expression in motor neurons under *D42-Gal4* led to a reduction in the number of Brp positive puncta, indicating a reduction in active zones of NMJs [t_(21)_ = 3.177, *p* = 0.0045, *t*-test] (Figure 4A). However, no significant reduction in levels of Syt, indicated by changes in Syt intensity, was detectable [t_(26)_ = 0.9060, *p* = 0.3733, *t*-test] (Figure 4B).

The overgrowth of boutons can lead to the formation of satellite boutons, which are additional boutons that bud off from the parent bouton or from the main arbor of NMJs (Dickman et al., 2006; O'Connor-Giles and Ganetzky, 2008). We quantified the number of satellite boutons as a measure of aberrant growth of the NMJs and observed that Q78 expression in motor neurons led to the appearance of numerous satellite boutons budding off from the main boutons of NMJs (*p* < 0.0001, Mann–Whitney *U*-test) (Figure 4C). Such satellite boutons were rarely seen in the NMJs of Q27 controls.

Proteostasis impairment due to polyQ aggregate accumulation can be a leading cause of neuronal dysfunction. One of the important proteostasis pathways is autophagy, and recent studies have shown that it is highly compartmentalized in neurons (Maday and Holzbaur, 2014, 2016). Several lines of research indicate presynaptic autophagy to be important for the normal functioning and development of synapses (Vijayan and Verstreken, 2017; Kuijpers et al., 2021). Apart from physiological substrates generally degraded *via* the general autophagy pathway, pathophysiological substrates, such as amyloid fibrils, hyperphosphorylated tau tangles, and polyQ aggregates, are also known to be degraded *via* the selective autophagy pathway called aggrephagy (Ravikumar et al., 2002; Rubinsztein, 2006; Yamamoto and Simonsen, 2011; Lamark and Johansen, 2012). However, overwhelming levels of aggregate accumulation in neurons can lead to severe impairment in proteostasis pathways, including the ubiquitin-proteasome system and autophagy (Nassif and Hetz, 2012; Hipp et al., 2014; Klaips et al., 2018; Thibaudeau et al., 2018). We, therefore, asked if the accumulation of Q78 aggregates in motor neuron cell bodies could possibly lead to basal-level autophagy impairment and probed for endogenous levels of Atg8a, the ortholog of LC3 in *Drosophila*. We observed that, in comparison to Q27 expressing larvae, Q78 expression in motor neurons led to a significant reduction in the levels of endogenous Atg8a, indicating a possible block in the pathway [t_(22)_ = 2.422, *p* = 0.0241, *t*-test] (Figure 4D). Taken together, these results suggest that Q78 expression in motor neurons leads to functional changes in the glutamatergic synapses of *Drosophila* NMJs.

### Overexpression of Atg8a in Motor Neurons Leads to Rescue of Defects in Glutamatergic Synapses

**Figure 5:**
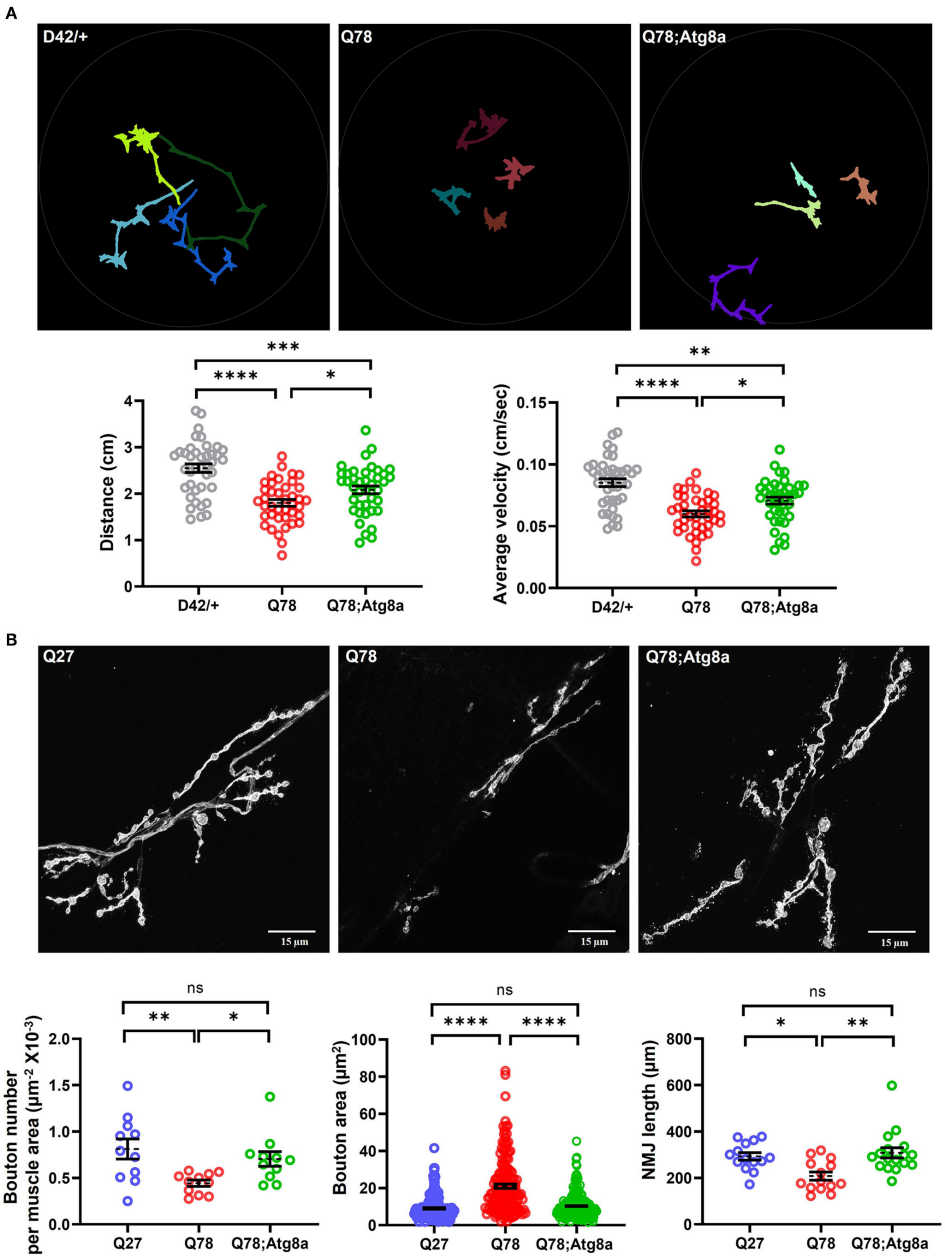
The behavioral and morphological defects in larval glutamatergic synapses are rescued upon Atg8a overexpression. **(A)** Path diagrams for indicated larvae monitored in 1% charcoal agar plates and quantification of total distance traversed and average velocity of the driver-only control larvae (D42/+) vs. pathogenic Q78 and rescue Q78; Atg8a larvae. *n* = 40 larvae; one-way ANOVA; *post-hoc* Tukey's multiple comparison test; *p < 0.05; **p < 0.01; ***p < 0.001; ****p < 0.0001; ns, non-significant. Error bars represent mean ± SEM. **(B)** Representative images of NMJs of indicated larvae from muscle 6/7 of abdominal hemisegment A2. Quantification of the number of boutons per muscle area (*n* > 11 NMJs), bouton area (*n* > 160 boutons), and NMJ arbor length (*n* > 12 NMJs). Quantifications for the number of boutons per area of muscle were done on hemisegment A2. Quantifications for the bouton area and NMJ arbor length were done on NMJs of muscle 6/7 of abdominal segments A4-A6. One-way ANOVA; *post-hoc* Tukey's multiple comparison test; *p < 0.05, **p < 0.01, ***p < 0.001, ****p < 0.0001. ns, non-significant. Error bars represent mean ± SEM.

Previous studies have shown that restoring the imbalance in proteostasis pathways either by genetic or pharmacological means can reduce the toxicity caused by the accumulation of mutant proteins in the central nervous system (Simonsen et al., 2008; Ordonez et al., 2012; Yi et al., 2013; Charif et al., 2022). We thus wanted to understand if genetic overexpression of Atg8a specifically in motor neurons, can lead to a rescue of the behavioral and synaptic defects in the ATXN3 polyQ model. To assess for rescue in behavioral deficits, we performed the locomotion assay at the larval stage. *D42-Gal4* served as the control, while *D42-Gal4* > *UASQ78* and *D42-Gal4* > *UASQ78; UASAtg8a* were the experimental genotypes. We observed that Q78 expression under *D42-Gal4* led to a significant reduction in average velocity in comparison to control. This defect was significantly rescued upon overexpression of Atg8a in the background of mutant Q78 under *D42-Gal4* [genotype, *F*_(2, 117)_ = 20.45, *p* < 0.0001, ANOVA; D42/+ vs. Q78 (*p* < 0.0001), D42/+ vs. Atg8a; Q78 (*p* = 0.0011), Q78 vs. Atg8a; Q78 (*p* = 0.0204), Tukey's multiple comparison] (Figure 5A, right). Similarly, the total distance traveled by the larvae was also rescued on Atg8a overexpression [genotype, *F*_(2, 117)_ = 20.53, *p* < 0.0001, ANOVA; D42/+ vs. Q78 (*p* < 0.0001), D42/+ vs. Atg8a; Q78 (*p* = 0.0004), Q78 vs. Atg8a; Q78 (*p* = 0.0477), Tukey's multiple comparison] (Figure 5A, left).

Since we observed a rescue in the larval crawling behavior upon overexpression of Atg8a, we wanted to understand if there were significant changes in the morphology and/or function of glutamatergic synapses as well. To answer this question, we imaged the FITC-HRP-labeled NMJs of muscle 6/7 and scored for aberrant bouton phenotype rescue upon overexpression of Atg8a in the background of Q78. We observed that the number of boutons per NMJ was significantly increased upon overexpression of Atg8a as compared to Q78-expressing larvae [genotype, *F*_(2, 30)_ = 3.730, *p* = 0.0080, ANOVA; Q27 vs. Q78 (*p* = 0.0003), Q27 vs. Atg8a; Q78 (*p* = 0.7325), Q78 vs. Atg8a; Q78 (*p* < 0.0001), Tukey's multiple comparison) (Figure 5B, left). Furthermore, the NMJ arbor length was also increased significantly upon overexpression of Atg8a, comparable to controls [genotype, *F*_(2, 42)_ = 7.569, *p* = 0.0016, ANOVA; Q27 vs. Q78 (*p* = 0.0072), Q27 vs. Atg8a; Q78 (*p* = 0.6093), Q78 vs. Atg8a; Q78 (*p* = 0.0678), Tukey's multiple comparison] (Figure 5B, right). The bouton area of NMJs in these Atg8a-overexpressed larvae showed a comparable area of boutons similar to controls in comparison to the increased bouton area of Q78-expressing larvae [genotype, *F*_(2, 551)_ = 77.17, *p* < 0.0001, ANOVA; Q27 vs. Q78 (*p* < 0.0001), Q27 vs. Atg8a; Q78 (*p* = 0.4190), Q78 vs. Atg8a; Q78 (*p* < 0.0001), Tukey's multiple comparison) (Figure 5B, center). We next assessed the levels of Ref(2)P (fly ortholog of p62), a ubiquitin-binding scaffold protein involved in the capture and degradation of cargo, and a substrate of the autophagy pathway. We observed that, in larvae co-expressing Q78 and Atg8a, the intensity of p62 puncta was reduced compared to those expressing Q78 alone [t_(14)_ = 3.289, *p* = 0.0054, *t*-test] (Supplementary Figure 3), suggesting autophagy-dependent degradation of p62.

Altogether, these results suggest that overexpression of Atg8a in Q78 mutants significantly rescued the phenotypes observed both at the behavioral level and at the cellular level. Thus, we show that genetic overexpression of the core autophagy protein Atg8a has the potential to rescue behavioral and cellular defects arising out of glutamatergic synapse dysfunction in a *Drosophila* model of MJD.

## Discussion

Synapses are important communication centers of neuronal networks through which information in the form of electrical or chemical cues is transferred. Synaptic dysfunction is known to be an early sign of many neurodegenerative disorders, such as Alzheimer's, Parkinson's, Huntington's, and prion disease (Graveland et al., 1985; Kitamoto et al., 1992; Scheff and Price, 2003; Coleman et al., 2004; DiProspero et al., 2004; Compta and Revesz, 2021). Such dysfunction may be a result of various insults, including the accumulation of misfolded and aggregated proteins, thereby hampering the normal functioning of synapses (Scott et al., 2010; Zhou et al., 2017; Ghiglieri et al., 2018). Polyglutamine-(polyQ) expansion diseases are a group of neurodegenerative conditions, encompassing nine heritable genetic disorders. The pathogenicity of these diseases is attributed to unstable CAG trinucleotide repeats within protein-coding regions, resulting in the formation of polyQ repeat-containing proteins (Riley and Orr, 2006).

Various studies have shown the involvement of polyQ repeats in ATXN3 in the pathogenesis and disease progression of the central nervous system. However, peripheral nervous system dysfunction is relatively unexplored (Colomer Gould et al., 2007; Schmidt et al., 2019; Wiatr et al., 2021). Human neurodegenerative diseases have been modeled in relatively simpler invertebrates, such as *D. melanogaster* and *Caenorhabditis elegans*, as these systems serve as excellent *in vivo* models to understand mechanisms of disease pathology, owing to homology to the human genome, and the vast number of tools available to genetically manipulate these organisms (Lu and Vogel, 2009; Caldwell et al., 2020). Using *Drosophila* as the model organism, Warrick et al. showed that targeted expression of the mutant Ataxin-3 in different cell types led to neurodegeneration. Particularly, neuronal cells were most susceptible to degeneration (Warrick et al., 1998). While their study explored the effects of expressing MJDtr-Q78 under different *GAL4* drivers (*gmr-GAL4, elav-GAL4, 24B-Gal4, and dpp-GAL4*), in our study, we focused on the dysfunction of synapses associated with neurodegenerative diseases. We used this model as a tool to understand synaptopathy associated with Spinocerebellar Ataxia Type-3, with a particular focus to assess if modulating proteostatic machinery in the synapses can lead to a rescue of the defects. While expressing MJDtr-Q78 (strong) under *elav-GAL4* is lethal, our study has utilized targeted expression of MJDtr-Q78 (strong) in the motor neurons. These flies do not show lethality in the early stages, allowing us to utilize larval NMJs as model glutamatergic synapses to understand the dysfunction associated with SCA3, thus enabling a narrow dissection of behavioral and physiological defects and their causation.

### Behavioral and Physiological Functions Are Affected Due to Expression of Pathogenic Q78 in Glutamatergic Motor Circuits

In our study, we were able to characterize the defects at the behavioral level in the larval and adult stages. With the well-established locomotion assay, we could characterize various aspects of locomotory defects in the larval stage as well as other behavioral defects associated with mutant Q78 expression in motor neurons in the adult stage. Our results are in line with previous studies, which have shown similar behavioral defects in other neurodegenerative models of *Drosophila* larvae and adults, owing to aggregate protein expression and, hence, cellular malfunctioning (Mershin et al., 2004; Mhatre et al., 2014; Wu et al., 2017; Delfino et al., 2020).

### Synapse Morphology and Health Are Compromised by the Presence of Pathogenic ATXN3 in Motor Neuron Soma

The rhythmic movements of the larvae have been attributed to concerted actions of motor neurons, sensory neurons, and interneurons (Kohsaka et al., 2012). Indeed, electrophysiological recordings from the larval NMJs during wave contractions of the larval body have shown concurrent rhythmic patterns (Fox et al., 2006; Imlach et al., 2012; Ruiz et al., 2013). Thus, any dysfunction in this circuitry can lead to locomotion impairment. We explored the possibility of glutamatergic synapses becoming dysfunctional upon expression of Q78. We found that mutant Q78 accumulated as aggregates in the ventral nerve cord (VNC) unlike in controls. This finding suggests the possibility of the truncated form of MJDtrQ78 containing 78 polyQ repeats being retained in the nucleus, as reported by several groups (Lee et al., 2004; Bichelmeier et al., 2007; Macedo-Ribeiro et al., 2009). Accumulation of mutant Q78 led to morphological changes in the NMJs. A possible reason could be relative transcriptional changes in proteins or modification in the levels of proteins/phosphoproteins required for synapse maintenance and/or functioning, as has been reported by various groups for ATXN3 models (Ramani et al., 2017; Wiatr et al., 2021). Moreover, the heterogeneity in terms of defects in the NMJs might be attributed to differences in expression of the driver *D42-Gal4*, which would require further investigation.

It is known that morphological changes in the NMJs are often correlated with functional defects as well, which might lead to behavioral deficits (Sleigh et al., 2014; Cappello and Francolini, 2017). We further assessed for functional defects in the boutons of NMJs. Synaptic vesicles loaded with neurotransmitters are brought close to the presynaptic membrane of the *Drosophila* NMJ by the concerted actions of presynaptic proteins, such as Bruchpilot (Brp) and another vesicular SNARE, Synaptotagmin (Syt), thus leading to the docking and fusion of these vesicles and concomitant release of glutamate (Quiñones-Frías and Littleton, 2021; Sauvola and Littleton, 2021). Brp and Syt are also essential at the *Drosophila* neuromuscular junctions for the clustering of calcium channels (Kittel et al., 2006; Wagh et al., 2006). *Brp* null mutants (*brp*^69^) have been shown to be defective in the fusion of glutamate-containing neurotransmitter vesicles in *Drosophila* NMJs (Paul et al., 2015). Thus, any changes in the levels of Brp and Syt may alter the functioning of glutamatergic synapses. Our results showed that Q78 expression in larval motor neurons led to a reduction in Brp-containing active zones but no change in Syt levels. The abundance of Brp positively correlates with neurotransmitter release (Matz et al., 2010; Weyhersmüller et al., 2011; Ehmann et al., 2014). However, in our study, it might be possible that the mutant Q78 NMJs are defective in releasing glutamate, thus correlating with behavioral deficits.

Satellite boutons are often characteristic of endocytic mutants, such as *endo* as well as mutants of the BMP signaling pathway and actin regulation. These supernumerary boutons are usually functional boutons (unlike other overgrowth phenotypes, such as “ghost” boutons) since they contain Brp and Synapsin, which are in apposition to postsynaptic GluR and Dlg (Dickman et al., 2006). Our observation of the appearance of numerous satellite boutons in Q78 mutants might indicate defects in the endocytic pathway as well. The appearance of satellite boutons could also be a compensatory mechanism against functional defects in the NMJs. Future studies would be required to understand the underlying cause. Our results thus show changes in the physiology and health of glutamatergic synapses in mutant Q78 *Drosophila* NMJs. Such changes in NMJs have been previously reported in several neurodegenerative disorders across various models (Menalled et al., 2003; Dupuis and Loeffler, 2009; Steinert et al., 2012; Pratt et al., 2015; Rodríguez Cruz et al., 2020; Alhindi et al., 2021).

### Harnessing Proteostatic Pathways Can Mitigate the Toxicity of Malformed ATXN3

In synapses, fast protein turnover is a prerequisite for the proper functioning of synaptic vesicle cycles, which might exceed 100 Hz in some cases (de Kock and Sakmann, 2008). Such a turnover might not be possible in cases of long motor neurons where the main site of protein synthesis (soma) is often separated from the distal ends (synapses) by over a meter in length. Thus, local proteostatic mechanisms, such as autophagy and the ubiquitin-proteasome system, are often required to mediate the fast turnover and maintenance of these synapses (Ding and Shen, 2008; Gorenberg and Chandra, 2017; Vijayan and Verstreken, 2017; Andres-Alonso et al., 2021). Any impairment in these pathways, for example, due to aggregate accumulation as reported in the case of many neurodegenerative disorders, can lead to synaptopathies (Bridi and Hirth, 2018). Studies pertaining to the impaired autophagy pathway in MJD have shed light upon the role of ATXN3 as a regulator of this pathway. Ashkenazi and colleagues have shown that the polyQ domain of wild-type ATXN3 is important for regulating autophagy by interacting with beclin1, preventing its degradation *via* UPS. However, other polyQ proteins or mutant ATXN3 itself, harboring long polyQ repeats, competes for this interaction of wild-type ATXN3 with beclin1, thus leading to impairment in autophagy by mediating the degradation of beclin1 (Ashkenazi et al., 2017). We also detected impairment in the autophagy pathway as indicated by a reduction in autophagosome numbers in fly NMJs. Future studies will reveal more details of the mechanism of impairment upon Q78 expression in the NMJs. Several previous studies have suggested that restoring the proteostasis imbalance *via* genetic or pharmacological means can have therapeutic value (Corti et al., 2020; Joshi et al., 2020; Bastien et al., 2021). Overexpression of Atg8a has been shown to promote longevity and reduce age-induced oxidative stress in adult flies (Simonsen et al., 2008). Therefore, we examined whether overexpression of Atg8a in *Drosophila* motor neurons could suppress synaptic dysfunction caused by the presence of mutant ATXN3. Indeed, our results indicate that tissue-specific overexpression of one of the core autophagy proteins, Atg8a, in the motor neurons of *Drosophila* facilitated a rescue in morphological and functional defects in the glutamatergic synapses of ATXN3 models, which is further recapitulated in the improvement of behavioral defects at the larval stage. The NMJ morphology is rescued in terms of bouton number. However, we do see a satellite bouton phenotype similar to Q78, which might explain the partial rescue in the locomotory behavior of the larvae. Co-expression of Atg8a with Q78 also resulted in lower levels of Ref(2)P in the NMJs, implicating the involvement of the autophagy pathway.

Our study sheds light on the pathogenesis of glutamatergic synapses *in vivo* in MJD. The defects observed in the glutamatergic synapses at the cellular and behavioral levels are consistent with other aggregate-induced neurodegenerative models (Jacobsen et al., 2006; Kohsaka et al., 2012; Hall et al., 2015; Maulik et al., 2017; Rattray et al., 2017; Caldwell et al., 2020). The genetic amenability of this model allows for quick forward genetic and modifier screens to identify potential mediators of synaptopathies. Furthermore, the observed rescue of defects demonstrates the usefulness of this model in screening for pharmacological and genetic candidates with therapeutic potential for the treatment of synaptopathies.

## Data Availability Statement

The raw data supporting the conclusions of this article will be made available by the authors, without undue reservation.

## Author Contributions

AC contributed to the conceptualization of the original study, performed the larval behavioral experiments, larval NMJ dissections, larval NMJ image acquisition, analysis, and manuscript writing. AS performed adult fly experiments, larval brain dissection, image acquisition of larval brain, statistical analysis, and data curation. VS and RM conceptualized and supervised the study. All authors contributed to the article and approved the submitted version.

## Funding

This work was supported by the Department of Biotechnology (DBT) grant in Life Sciences Research, Education and Training at JNCASR (BT/INF/22/SP27679/2018), the Intramural funds from JNCASR to RM and VS, and the JNCASR doctoral fellowships to AC and AS.

## Conflict of Interest

The authors declare that the research was conducted in the absence of any commercial or financial relationships that could be construed as a potential conflict of interest.

## Publisher's Note

All claims expressed in this article are solely those of the authors and do not necessarily represent those of their affiliated organizations, or those of the publisher, the editors and the reviewers. Any product that may be evaluated in this article, or claim that may be made by its manufacturer, is not guaranteed or endorsed by the publisher.
