# Supplementary figures and images for: Glutamatergic Synapse Dysfunction in Drosophila Neuromuscular Junctions Can Be Rescued by Proteostasis Modulation
Source: Front Mol Neurosci. 2022 Jul 15;15:842772. doi: 10.3389/fnmol.2022.842772 (PMC9337869; doi:10.3389/fnmol.2022.842772)

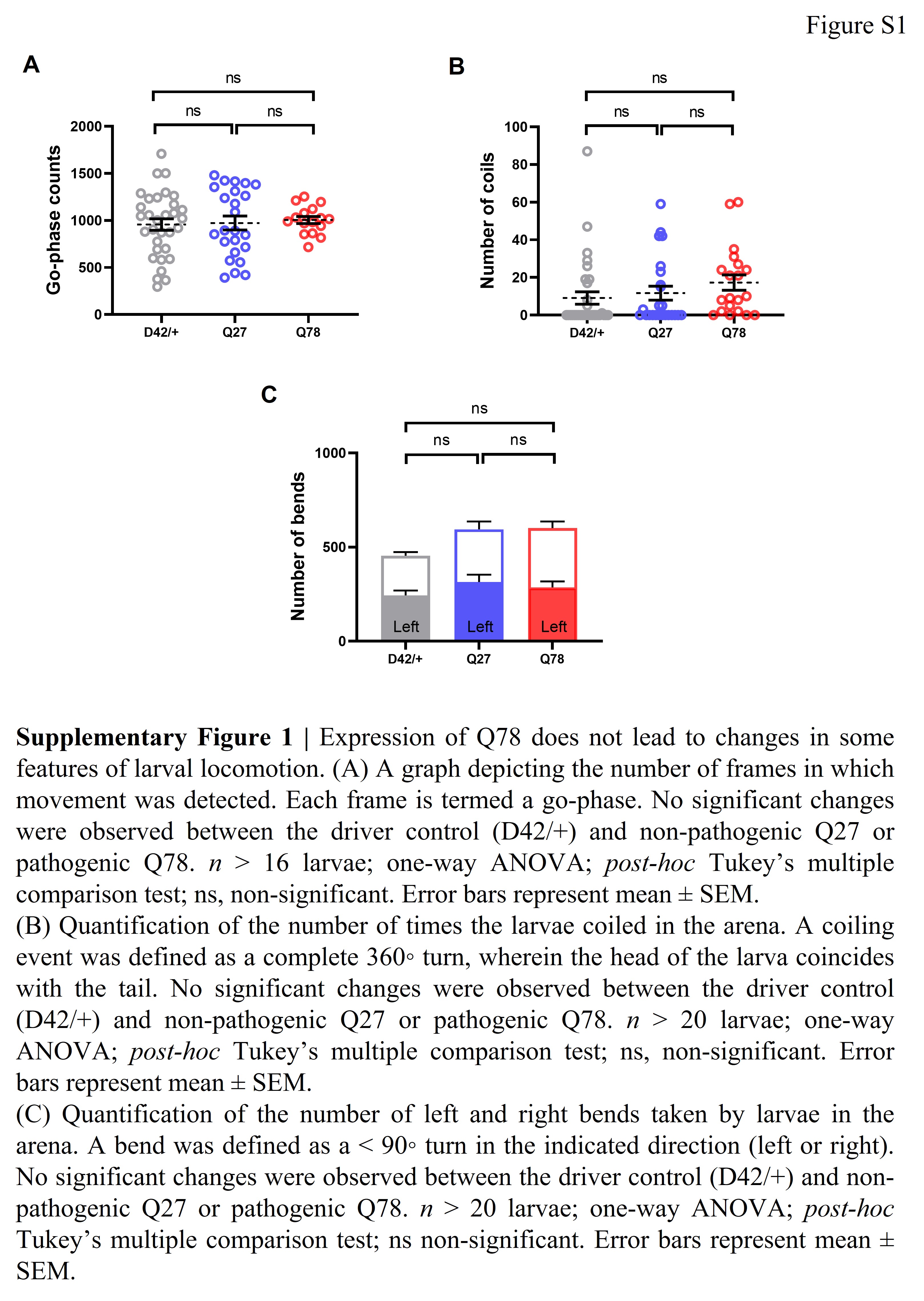

Supplement: Supplementary file 1 [file Image_1.jpg]

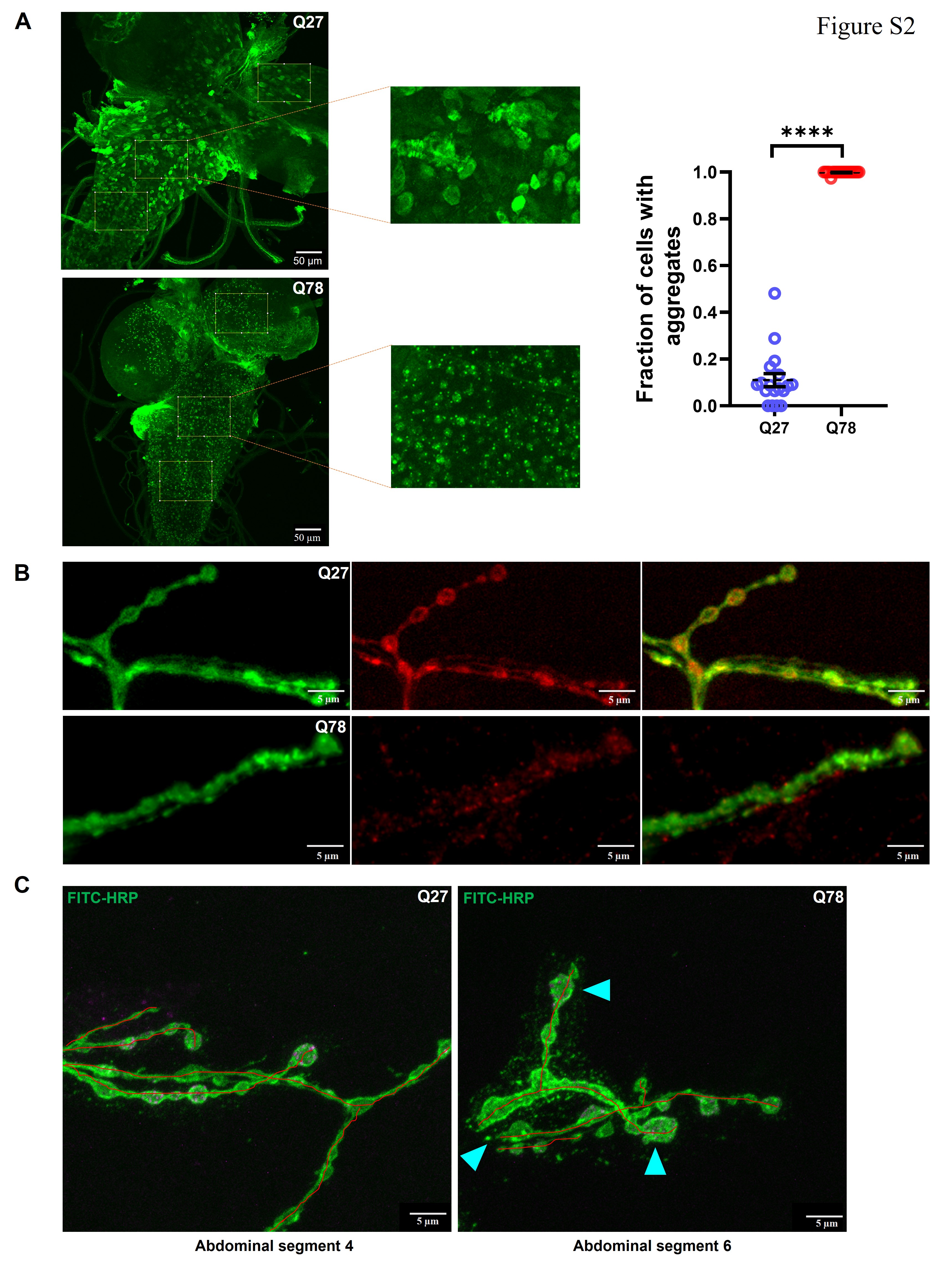

Supplement: Supplementary file 2 [file Image_2.jpg]

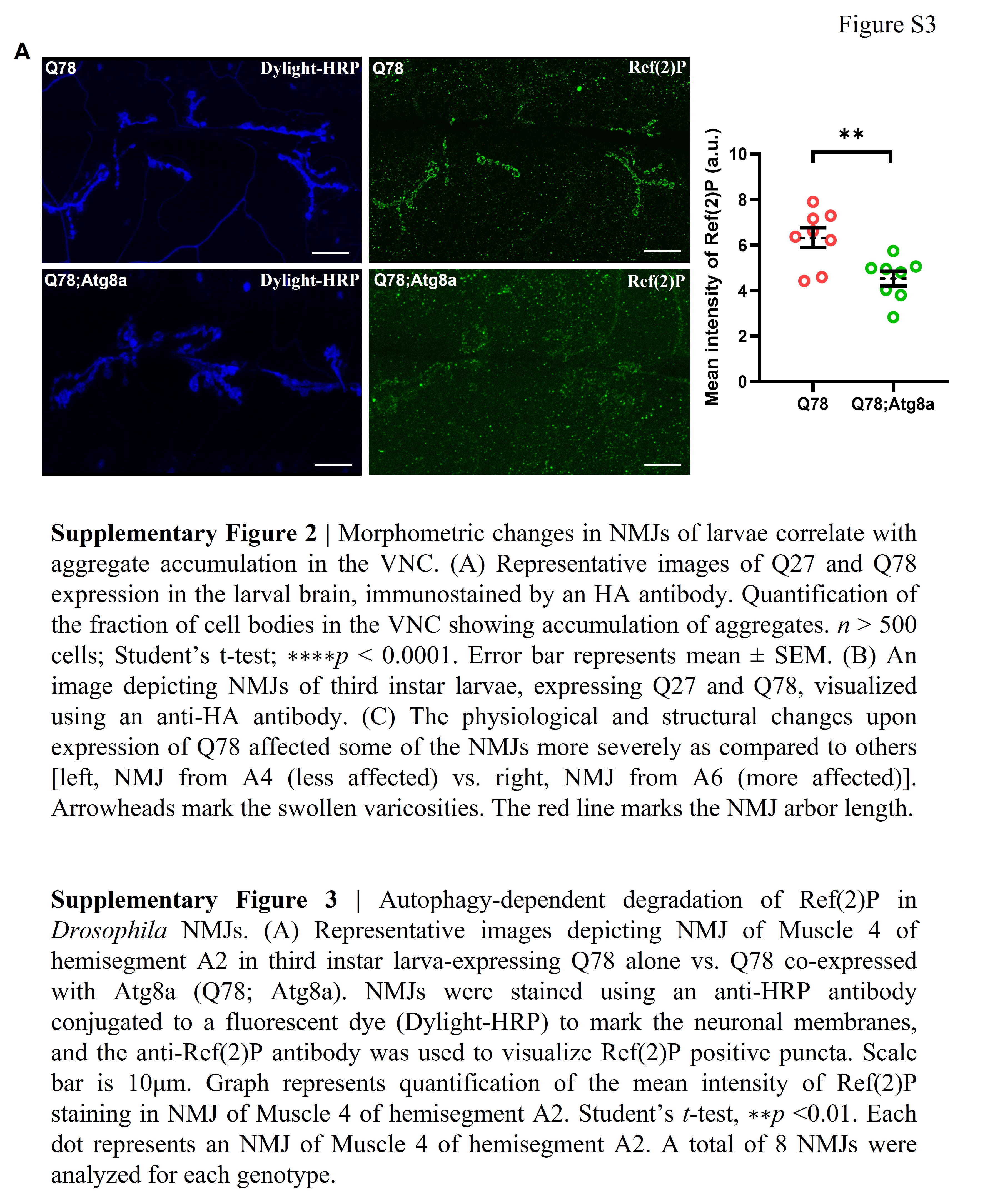

Supplement: Supplementary file 3 [file Image_3.jpg]
